# Meeting of January 21, 1898

**Published:** 1898-03

**Authors:** 


### MEETING OF JANUARY 21, 1898.

### CONGENITAL ABSENCE OF PECTORAL MUSCLE.

Dr. Taylor presented a patient who had been brought to him because of asymmetry of the upper part of the chest in front. The patient was a boy six weeks old. There was normal fullness of the right side while the left side where the pectoralis major should have been was much depressed. This was the fifth child of the mother, the others being healthy. Labor was normal. The child moved both arms equally well.

Dr. Whitman presented a boy sixteen years old with a similar condition. When five years old he had pigeon breast. On account of round shoulders and projecting scapulæ he had been brought to Dr. Whitman, who had found a defect of the right pectoralis major due, apparently, to congenital malformation, from which the boy had suffered little inconvenience, although he was left handed. The muscular deficiency had never been recognized. The clavicular part of the muscle was normal, but sternal part was represented simply by a fold of fibrous tissue which could be brought into prominence by certain motions of the arm. He had considered the possibility of poliomyelitis as a cause, but after seeing Dr. Taylor's patient, he thought the trouble was due to congenital malformation. He had seen a somewhat similar case in which absence of the right pectoral muscle had been accompanied by defective ribs, slight lateral curvature and webbed fingers.

Dr. Myers had the day before seen a patient affected with wry-neck from general shortening of the muscles of the right side. There was also on both sides a very marked depression between the inner border of the deltoid and outer border of the pectoralis major, apparently due to absence or defect of the clavicular part of the large pectoral muscle.

Dr. Sayre said that in a boy who had been brought to him for pigeon breast he had found the scapulæ very prominent and an almost complete absence of the right pectoralis major. The disability was not serious. He had referred the condition to an attack of poliomyelitis at the age of three, of which he had obtained what seemed to be a credible history. The case might have been, however, an instance of congenital defect of the muscle.

Dr. Elliott did not think that it was possible to exclude poliomyelitis in either of the patients shown. He had seen patients in whom a muscular defect in other parts of the body, at first thought to be congenital, had been found to be the result of poliomyelitis. He thought that cases were not uncommon in which the effects of the disease were limited to a single muscle or even to a part of a muscle.

Dr. Taylor said that in the case of a baby it would be necessary to assume that the disease had been in utero, as the condition of the muscle had been present from birth, and the atrophy was so complete that it suggested the absence of development rather than paralysis. If there had been paralysis it must have been at the very beginning of the development of the muscle, since paralyzed muscles retained their fulness for a considerable time.

### DEFORMITIES FOLLOWING TYPHOID FEVER.

Dr. W. R. Townsend presented a boy 19 years of age who had complained of spinal pain and stiffness since recovery from typhoid fever last February. The vertical column was very rigid with a slight curve towards the right in the lower dorsal region, and a posterior curve of the lower dorsal and the entire lumbar region. There were also a number of swellings distinctly connected with the bone in different parts of the body resembling the cold abscesses of tubercular subjects and syphilitic nodes. They were not very soft, and there was no fluctuation. The general health had been poor since the fever. Parsons, of Johns Hopkins University, had described such swellings as appearing several months after typhoid fever. He had found in them the typhoid bacillus, the staphylococcus and the bacillus coli communis and had advocated total extirpation of these foci.

Dr. Sayre thought the boy might be suffering from hereditary syphilis which had first made its appearance when his health was broken down by the attack of typhoid fever. If local treatment was necessary the foci might be incised and scraped and packed from the bottom. As the epiphysis is involved in several instances, enucleation would endanger the usefulness of the joints. He called attention to the girdle-mark, which is a pathognomonic sign of disease of the spine, and advised treatment as of an ordinary case of tuberculous disease of the spine.

Dr. V. P. Gibney advised that a trial of anti-syphilitic treatment be followed by general constitutional treatment, the administration of cod liver oil, etc. He could see no advantage likely to follow cutting out the foci. Spinal rigidity after typhoid fever was due to a mild periostitis about the points of exit of the nerves. He thought that forcible correction with anæsthesia would be excellent treatment in this case. He had seen a number of typhoid hips. One of them was under treatment by repeated forcible motion under anæsthesia followed by massage.

Dr. Whitman thought that the spinal deformity was the most important feature of the case, and that it required immediate correction. He said that the girdle-wrinkle was not caused by muscular spasm, but was simply a fold in the abdominal wall answering to the projection backwards which had taken the place of the normal lumbar lordosis.

Dr. Sayre said that he had noticed the girdle-wrinkle in many cases. It would be higher or lower, according to the location of the disease. It was due to muscular spasm which accompanied any muscle subject to irritation and joint inflammation. It was diagnostic of Potts' disease, and was present even when there was no appreciable projection.

Dr. Townsend said he had not thought seriously of taking the foci out, as to do so would, in nearly every instance in the patient in question, involve opening into a neighboring joint. He would put the boy upon anti-syphilitic treatment, and later would probably consider the other suggestions made.

### CONGENITAL MALFORMATION OF THE LOWER EXTREMITIES.

Dr. Gibney exhibited photographs of a boy 12 years old, whose limbs were normal above the knee, but remarkably deformed below. The right leg had no fibula, while the tibia was greatly curved. The foot had only three metatarsal bones, and two of the toes were webbed. The left leg was longer than the right, and the left foot was clubbed, and the bones very much atrophied. The astonishing part of it all was the way in which the boy walked. He got about very well, indeed. Deformity: Talipes equino-valgus right side, talipes equino-varus left side. It had been decided to amputate the feet in the anterior two-thirds and have the boy fitted with artificial legs.

### ABSCESSES WITH PERFORATION OF THE BLADDER.

Dr. Myers related the case of a boy 10 years old who had left hip disease, with many sinuses and waxy liver. A discharge of urine from a sinus in the inguinal region continued for two weeks. No pus was noticed in the urine. For a time there was pain in the lower part of the abdomen. The urine contained hyaline and granular casts, a few pus cells attached to casts, no sugar and a small amount of albumen. Specific gravity, 1010. The child was kept lying on the opposite side. Dr. Myers also related the case of a girl 15 years old who had many abscesses from disease of the left hip. An abscess appeared above Poupart's ligament on the right side, with abdominal pain. The muscles of the abdominal wall were rigid. Large quantities of pus were painfully passed with the urine. The abscess, after extending towards the left, ruptured, and with the escape of a quart of purulent fluid the pus disappeared from the urine. Both of the patients recovered from the perforation. In the first patient the flow was from the bladder outward, in the second from the abscess into the bladder. He also recalled two cases in which there was intestinal perforation, with discharge of intestinal contents through the sinus. Both patients speedily died.

Dr. Townsend recalled a case of psoas abscess in which pus passed for three years through a perforation in the rectum.

Dr. Sayre recalled a case of hip disease in an adult in which an abscess discharged through the bladder. The patient survived the complication ten years, and is still alive. In another patient, in whom both hips were diseased, on one side there was perforation into the intestine with escape of gas from an external sinus. This hip recovered with the motion, while the other hip, in which there was no abscess, recovered with anchylosis.

